# An Efficient Protocol for the Synthesis of Quinoxaline Derivatives at Room Temperature Using Recyclable Alumina-Supported Heteropolyoxometalates

**DOI:** 10.1100/2012/174784

**Published:** 2012-03-12

**Authors:** Diego M. Ruiz, Juan C. Autino, Nancy Quaranta, Patricia G. Vázquez, Gustavo P. Romanelli

**Affiliations:** ^1^Cátedra de Química Orgánica, Facultad de Ciencias Agrarias y Forestales, Universidad Nacional de La Plata, Calles 60 y 119, La Plata B1904AAN, Argentina; ^2^Facultad Regional San Nicolás, Universidad Tecnológica Nacional (UTN), Colón N-332, San Nicolás, Buenos Aires 2900, Argentina; ^3^Centro de Investigación y Desarrollo en Ciencias Aplicadas “Dr. J.J. Ronco” (CINDECA), Departamento de Química, Facultad de Ciencias Exactas, UNLP-CCT-CONICET, Calles 47 No. 257, La Plata B1900AJK, Argentina

## Abstract

We report a suitable quinoxaline synthesis using molybdophosphovanadates supported on commercial alumina cylinders as catalysts. These catalysts were prepared by incipient wetness impregnation. The catalytic test was performed under different reaction conditions in order to know the performance of the synthesized catalysts. The method shows high yields of quinoxaline derivatives under heterogeneous conditions. Quinoxaline formation was obtained using benzyl, *o*-phenylenediamine, and toluene as reaction solvent at room temperature. The CuH_2_PMo_11_VO_40_ supported on alumina showed higher activity in the tested reaction. Finally, various quinoxalines were prepared under mild conditions and with excellent yields.

## 1. Introduction

Quinoxaline derivatives are a very important class of nitrogen-containing heterocycles (containing benzene and pyrazine rings in their structure), as they constitute useful intermediates in organic synthesis. This substructure plays an important role as a basic skeleton for the design of a number of heterocyclic compounds with different biological activities, making this type of compounds important in the fields of (a) medicine: antitumor, anticonvulsant, antimalarial, anti-inflammatory, antiamoebic, antioxidant, antidepressant, antiprotozoal, antibacterial, and anti-HIV agents [1–10] and (b) technology: fluorescent dying agents, electroluminescent materials, chemical switches, cavitands, and semiconductors [11–17]. Quinoxalines are important in the pharmaceutical industry, with antibiotics such as echinomycin, levomycin, and actinoleutin having quinoxaline as part of their structure [18].

A number of synthetic strategies are known for the preparation of substituted quinoxalines. The classic method for quinoxaline preparation is the condensation of a 1,2-dicarbonylic compound with a 1,2-diamino compound. In general, this procedure needs high temperature, the use of a strong acid catalyst, and long reaction times [19]. Other strategies involve oxidative coupling of epoxides and 1,2-diamines [20], cyclization of aryl amino oximes and *α*-dicarbonyl compounds [21], and tandem oxidation of *α*-hydroxyl ketones [18].

A variety of catalysts were tested in these reactions such as acetic acid [22], iodine [23], CuSO_4_·5H_2_O [24], nickel nanoparticles [25], gallium(III)triflate [26], montmorillonite K10 [27], ionic liquids [28], Nano-TiO_2_ [29], sulfated TiO_2_ [30], Pd(OAc)_2 _[19], RuCl_2_-(PPh_3_)_3_-2,2,6,6-tetramethylpiperidine 1-oxyl(TEMPO) [19], MnO_2_, [19], Al_2_O_3_ [31], zirconium(IV)-modified silica gel [32], nanocrystalline CuO [33], cerium(IV) ammonium nitrate [34], iron exchanged molybdophosphoric acid [35], silica-bonded S-sulfonic acid [36], and sulfamic acid/MeOH [37]. Different reaction media were used to perform this synthesis such as the use of acetonitrile [23] or DMSO [38] as solvents, or even cleaner ways as the solvent-free reaction [31, 39], with various ways to give energy to the substrate, such as microwave radiation [31, 39], ultrasound [33], or even room temperature [30, 36, 37].

The need for greener techniques leads to using different environmentally friendly reaction conditions; among them the replacement of pollutant inorganic acid catalysts, such as sulfuric or hydrochloric acids with reusable solid acids and the use of room temperature avoiding media heating, is yet very necessary. The application of solid acids in organic transformation has an important role, because they have many advantages such as ease of handling, decreased plant corrosion, and more environmentally safe waste disposal procedures [36].

HPAs are molecular arrangements with remarkable and diverse electronic and molecular structures, which lead to their application in different areas such as medicine and materials science, among others; among the various possible HPA structures, the Keggin-type primary structure deserves to be mentioned, due to its widely reported applications [40]. As part of a research project to develop environmentally friendly organic reactions, we used different HPAs in various preparative reactions, under greener conditions such as the synthesis of coumarins [41], flavones and chromones [42], and N-sulfonyl-1,2,3,4-tetrahydroisoquinolines [43] among others.

As part of our ongoing research on the development of alternative synthetic procedures for the synthesis of biologically active heterocyclic compounds and the use of green chemistry techniques in organic synthesis, herein we report a simple and efficient method for the preparation of quinoxaline derivatives using heteropolyacids HPMo_11_VFeO_40_ and H_2_PMo_11_VCuO_40_ as catalysts. 

## 2. Materials and Methodology

### 2.1. General

All reagents were purchased from Merck and Aldrich and used without further purification. All the reactions were monitored by TLC on precoated silica gel plates (254 mm). Flash column chromatography was performed with 230–400 mesh silica gel. All the yields were calculated from pure products. All the products were identified by comparison of physical data (mp, TLC, NMR) with those reported. Melting points of the compounds were determined in sealed capillary tubes and are uncorrected. ^13^C NMR and ^1^H NMR spectra were recorded at room temperature on Varian-200 spectrometers using TMS as internal standard. Entries and target compounds have the same number.

### 2.2. Catalyst Preparation

#### 2.2.1. Synthesis of Catalyst

HPMo_11_VFeO_40_ (FeMoVP) and H_2_PMo_11_VCuO_40_ (CuMoVP) (specific surface area of the heteropolyacid with Keggin structure, from 3 to 10 m^2^/g) were prepared by a hydrothermal synthesis method [44, 45].

#### 2.2.2. Synthesis of Supported Catalysts

Commercial alumina (Akzo) (specific surface area, 282 m^2^/g; mean pore diameter, 4.2 nm; pore volume, 0.58 cm^3^/g) as cylinders was used as support. The support was used without further treatment. The supported catalysts were obtained by incipient wet impregnation of MPV solid using ethanol as solvent. The concentration of the impregnation solution was 120 gMo/l MPV solution. Then, the solids were dried at room temperature. Finally, the catalysts were thermally treated at 200°C for 6 h. The nomenclature is shown in Table 1.

### 2.3. Catalyst Characterization

**Figure 1:**
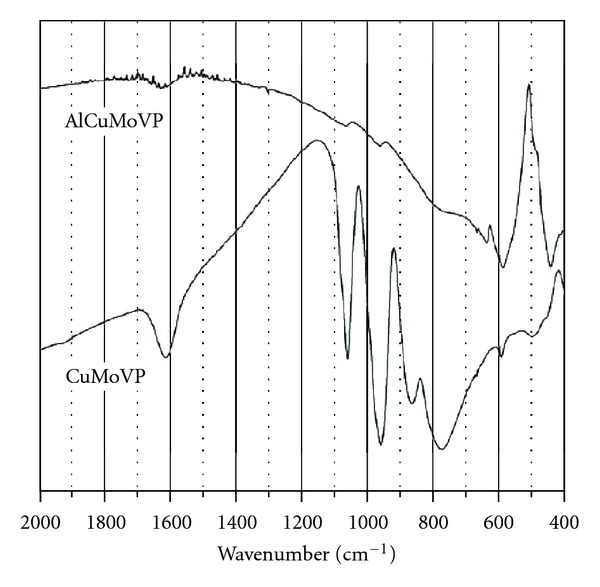
FT-IR spectra of bulk (CuMoVP) and supported AlCuMoVP.

In a previous paper, we reported the full characterization of both catalysts by diffuse reflectance spectroscopy (DRS), Fourier transformed infrared spectroscopy (FT-IR), optical and scanning electron microscopies, XRD analyses, and potentiometric titration.  Figure 1 shows the copper FT-IR spectra of bulk and alumina-supported catalysts [46]. 

### 2.4. Preparation of Quinoxaline General—Procedure

To a mixture of an o-phenylenediamine (1 mmol, 0.108 g) and 1,2-dicarbonyl compound (1 mmol) in toluene (8 mL), 0.1 g of MoVP catalyst was added and the mixture was stirred at room temperature. The progress of the reaction was monitored by TLC. After completion of the reaction, the insoluble catalyst was separated by filtration. The filtrate was dried over anhydrous Na_2_SO_4_. The solvent was evaporated, and the pure product was obtained. The products were purified by recrystallization from ethanol.

*2,3-Diphenylquinoxaline* (Table 6, entry 1). White needles, 92%, mp 127-128°C (lit. mp 127-128 [22]). ^1^H NMR (200 MHz, CDCl_3_): 8.20–8.10 (m, 2H), 7.82–7.70 (m, 2H), 7.60—7.35 (m, 10H). ^13^C NMR (50 MHz, CDCl_3_): 153.6, 141.3, 139.2, 130.0, 129.9, 129.4, 128.9, 128.4.

### 2.5. Recycling of the Catalyst

After reaction, the catalyst was filtered, washed thoroughly with toluene (2 × 3 mL), dried under vacuum, and reused for the next cycle, following the procedure described above.

## 3. Results and Discussion

**Scheme 1:**
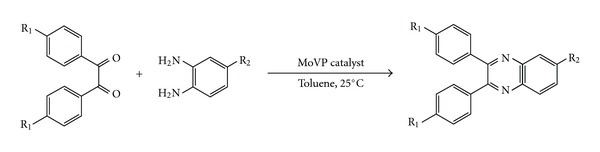
Synthesis of quinoxaline derivatives catalyzed by MoVP heteropolyoxometalates.

This work describes the application of a heterogeneous system for the preparation of quinoxalines in the presence of Keggin heteropolyoxometalates (AlCuMoVP and AlFeMoVP) as reusable catalyst. The quinoxaline synthesis involving the reaction of substituted *o*-phenylenediamines and 1, 2-diketones is illustrated in reaction Scheme 1.

Before attempting detailed catalytic work, a noncatalytic reaction between *o*-phenylenediamine (1 mmol), benzyl (1 mmol), and toluene (7 mL) was examined and it was observed that, under the experimental conditions (25°C, 2 h), no formation of quinoxaline was detected, indicating that from a practical point of view the reaction is not taking place in the absence of a catalyst (Table 2, entry 1). Similarly, no formation of quinoxaline was detected under the same reaction conditions using the support (Al) (Table 2, entry 2).

Table 1 lists the obtained results for quinoxaline yield using the two different catalysts considered (AlCuMoVP and AlFeMoVP). The experimental conditions were 100 mg of catalyst, 1 mmol of *o*-phenylenediamine, 1 mmol of benzyl, and 7 mL of toluene, reaction for 2 h at 25°C. Under these conditions, quinoxaline was obtained with a selectivity of 100% for both catalysts. The yields were 92% and 80%, respectively (Table 2, entries 3 and 4). The more active catalyst was used in the next experiments.

Table 3 displays the effect of the amount of catalyst (AlCuMoVP) on the yield of quinoxaline in the reaction. The experimental reaction conditions were *o*-phenylenediamine, 1 mmol; benzyl, 1 mmol; toluene, 7 mL, 120 min, 25°C, and a variable amount of AlCuMoVP catalyst (10, 50, 100, and 150 mg, resp.). It can be seen that the conversion of yields increased from 85% to 92% when the amount of AlCuMoVP increased from 50 to 100 mg (Table 3, entries 2 and 3). A further increase in the amount of AlCuMoVP (150 mg) caused a very slightly increase in azlactone yields (93%, Table 3, entry 4). Thus, 100 mg of AlCuMoVP is a suitable amount in this reaction. 

Table 4 shows the results for quinoxaline synthesis as a function of reaction time using AlCuMoVP catalyst at a reaction temperature of 25°C. The experimental reaction conditions were *o*-phenylenediamine, 1 mmol; benzyl, 1 mmol; AlCuMoVP, 100 mg; toluene, 7 mL and 25°C. It can be observed that the yields of azlactone increased with the reaction time up to 120 min and then stayed at a constant level.

The possibility of recycling the catalyst was examined. For this reason, the room temperature reaction of *o*-phenylenediamine and benzyl was studied in toluene in the presence of AlCuMoVP. When the reaction was complete, the mixture was filtered, the residue was washed with toluene and the recycled catalyst was reused in the next reaction. No appreciable loss of catalytic activity was observed after four cycles (Table 5 entry 4).

In order to estimate the possible catalyst solubilization, additional tests were performed. AlCuMoVP sample (100 mg) was stirred in toluene (7 mL) for 5 h, filtered and dried in vacuum till constant weight. Loss of mass was not detected. The refluxed toluene was used as solvent for attempting the reaction without adding the catalyst. After 5 h, quinoxaline was not detected and the starting material was quantitatively recovered.

**Scheme 2:**
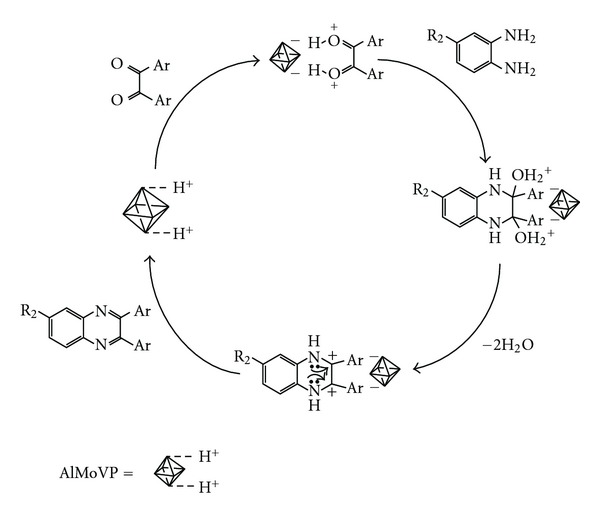
Proposed mechanism for the condensation reaction of 1,2-diamines with 1,2-dicarbonyl compounds in the presence of AlMoVP catalyst.

A plausible mechanism is rationalized in Scheme 2. As proposed by Niknam and Coworkers [36], the reaction follows a mechanism of acid-catalyzed condensation reactions, in our case with AlMoVP acting as a Brønsted acid, (1) coordination of a diketone to acid sites of AlMoVP, (2) the nucleophilic attack on the carbonyl intermediate, (3) dehydration to give a carbocation intermediate, and (4) elimination of a proton to give the quinoxaline product.

Encouraged by the remarkable results obtained with the above reaction and in order to show the generality and scope of this new protocol, we used various substituted 1,2-phenylenediamines and two diketones under the optimized conditions at room temperature (*o*-phenylenediamine: 1 mmol, benzyl: 1 mmol, toluene: 7–12 mL, and catalyst (AlCuMoVA): 100 mg); the results obtained are summarized in Table 6. All the reactions proceeded very cleanly at room temperature and no undesirable side reactions were observed, although the reaction time for a 100% conversion of substrates and reaction yields of products were highly dependent on the substituent. Results in Table 6 show that electron-donating groups at the phenyl ring of 1,2-diamine favored product formation (Table 6, entry 2). In contrast, electron-withdrawing groups such as chlorine and bromine slightly lowered the yields (Table 6, entries 3, 4, 8, and 9) with longer reaction times. 4-Nitro-2,3-diaminobenzene and 2,3-diaminopyridine also gave moderate yields (Table 6, entries 5 and 6).

## 4. Conclusions

In conclusion, supported molybdophosphovanadates, which can simply be prepared from commercially available and relatively cheap starting materials, are an efficient, thermally stable, and recoverable catalyst for the silylation of phenols and alcohols in toluene at ambient temperature. The present protocol provides a novel, efficient, and recyclable methodology for the preparation of quinoxalines in high yields with an easy workup procedure; the catalyst can be recovered and reused over several reaction cycles without considerable loss of reactivity. Moreover, this methodology introduces a practical and viable green technology for quinoxaline preparation. We are currently exploring further applications of this solid to other types of heterocycles.

## Figures and Tables

**Table 1:** Catalyst nomenclature.

Entry	Catalyst	Nomenclature-supported catalyst
1	Alumina cylinder	Al
3	FeHPMo_11_VO_40_	AlFeMoVP
4	Cu_2_H_2_PMo_11_VO_40_	AlCuMoVP

**Table 2:** Effect of catalyst silica on quinoxaline yields (%).

Entry	Catalyst	Yield^a^ (%)
1	None	—
2	Al	—
3	AlCuMoVP	92
4	AlFeMoVP	80

Reaction conditions: *o*-phenylenediamine, 1 mmol; benzyl, 1 mmol; toluene, 10 mL; 100 mg of supported catalyst; 120 min; 25°C.

^a^Isolated yield.

**Table 3:** Effect of amount of catalyst on quinoxaline yields (%).

Entry	Catalyst Amount (mg)	Yield^a^ (%)
1	10	43
2	50	85
3	100	92
4	150	93

Reaction conditions: *o*-phenylenediamine, 1 mmol; benzyl, 1 mmol; toluene, 10 mL; supported catalyst: AlCuMoVP; 120 min, 25°C.

**Table 4:** Effect of time of reaction on azlactone yields (%).

Entry	Reaction time (min)	Yield^a^ (%)
1	15	48
2	30	71
3	60	80
4	120	92
5	180	90

Reaction conditions: *o*-phenylenediamine, 1 mmol; benzyl, 1 mmol; toluene, 7 mL; supported catalyst: AlCuMoVP, 100 mg; 25°C.

**Table 5:** Catalyst reuse on 4-benzylidene-2-phenyloxazol-5-one yields (%).

Entry	Cycle	Yield^a^ (%)
1	0	92
2	1	90
3	2	90
4	3	88

Reaction conditions: *o*-phenylenediamine, 1 mmol; benzyl, 1 mmol; toluene, 10 mL; 100 mg of supported catalyst; 120 min; 25°C.

^a^Isolated yield.

**Table 6:** Preparation of quinoxalines using AlCuMoVP as catalyst.

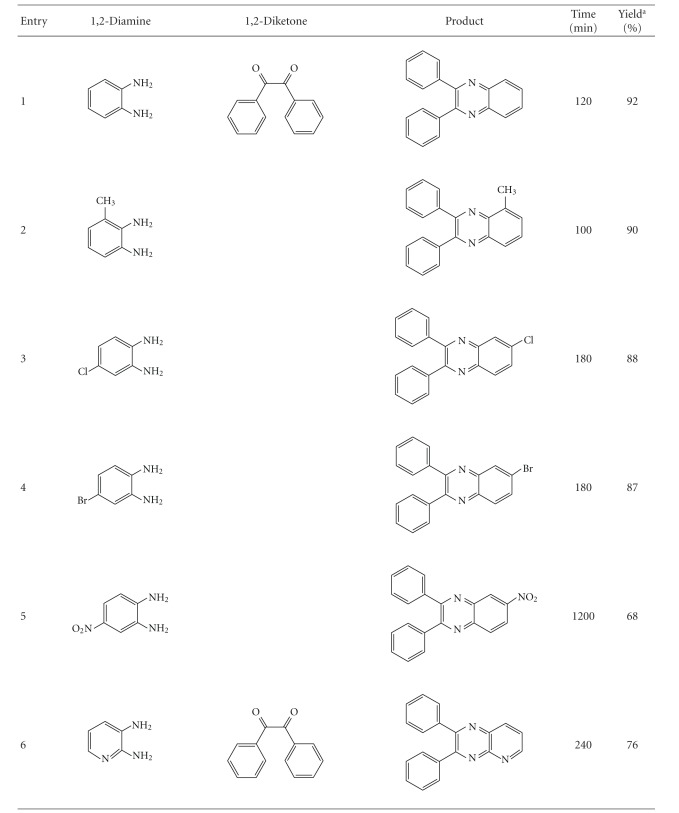 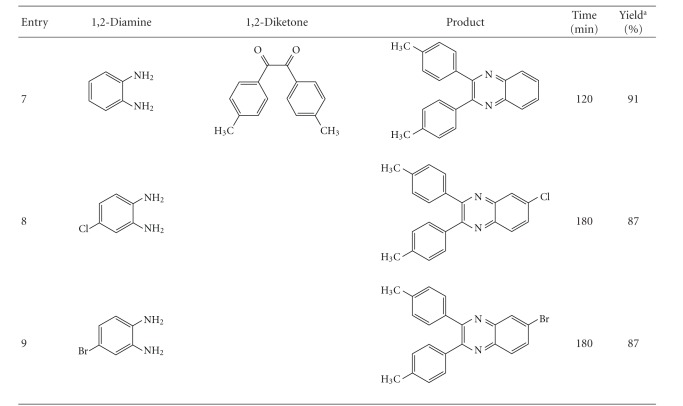

Reaction conditions: molar ratio of substrates: (1 : 1); catalyst: 100 mg. Reactions were run at 25°C. ^a^Isolated yield.
